# The Pharmaceutical Benefits Scheme 2003–2004

**DOI:** 10.1186/1743-8462-2-2

**Published:** 2005-01-12

**Authors:** Ken J Harvey

**Affiliations:** 1 School of Public Health, La Trobe University, 3086, Australia

## Abstract

The Pharmaceutical Benefits Scheme (PBS) grew by 8% in 2003–04; a slower rate than the 12.0% pa average growth over the last decade. Nevertheless, the sustainability of the Scheme remained an ongoing concern given an aging population and the continued introduction of useful (but increasingly expensive) new medicines. There was also concern that the Australia-United States Free Trade Agreement could place further pressure on the Scheme. In 2003, as in 2002, the government proposed a 27% increase in PBS patient co-payments and safety-net thresholds in order to transfer more of the cost of the PBS from the government to consumers. While this measure was initially blocked by the Senate, the forthcoming election resulted in the Labor Party eventually supporting this policy. Recommendations of the Pharmaceutical Benefits Advisory Committee to list, not list or defer a decision to list a medicine on the PBS were made publicly available for the first time and the full cost of PBS medicines appeared on medicine labels if the price was greater than the co-payment. Pharmaceutical reform in Victorian public hospitals designed to minimise PBS cost-shifting was evaluated and extended to other States and Territories. Programs promoting the quality use of medicines were further developed coordinated by the National Prescribing Service, Australian Divisions of General Practice and the Pharmacy Guild of Australia. The extensive uptake of computerised prescribing software by GPs produced benefits but also problems. The latter included pharmaceutical promotion occurring at the time of prescribing, failure to incorporate key sources of objective therapeutic information in the software and gross variation in the ability of various programs to detect important drug-drug interactions. These issues remain to be tackled.

## Review

This paper reviews the growth of the Pharmaceutical Benefits Scheme (PBS) during 2002–03; concerns about the sustainability of the Scheme, the government's response, a potential new threat that emerged and issues that remain to be tackled.

### The growth and sustainability of the PBS

From March 2003 to March 2004, a total of $5.8 billion was spent on prescription medicines subsidised under the PBS. Of this, $4.89 billion (84%) was paid by the Commonwealth, the remaining $0.91 billion through patient co-payments [1]. In comparison, in 2002–03, the Commonwealth spent $7.24 billion on public hospital services [2] and $8.17 billion on medical and diagnostic services (through Medicare benefits) [3]. Although the PBS is the smallest of these components of Commonwealth expenditure, it has the highest average annual growth rate over the last decade (around 12% pa), compared to 6% pa for public hospital services, and 5% pa for medical services. At these rates, by 2011 the Commonwealth would be spending more on subsidised pharmaceuticals than it would spend on either public hospital or medical services, and by 2022, more on pharmaceuticals than both public hospital and medical services together. Such projections make the sustainability of the PBS a major concern, especially given an aging population and the continued introduction of useful (but increasingly expensive) new medicines [4]. While the growth rate of the PBS has slowed over the last two years (10% during March 2002–03 and 8% from March 2003–04) the past history of PBS expenditure shows considerable fluctuations over the years. These fluctuations are caused by expensive but valuable new drugs coming onto the Scheme, more cost-effective generic drugs replacing older drugs whose patent has expired and administrative changes, such as increased patient co-payments, transiently reducing usage.

### The government's response

In 2003, as in 2002, the government proposed a 27% increase in PBS patient co-payments and safety-net thresholds in order to transfer more of the cost of the PBS from the government to consumers. Once again this measure was rejected by Labor and other opposition parties in the Senate because of concern that such increases would impact on equitable access to necessary medicines [5]. Regardless, the government continued to argue that without increased patient contributions (and patient restraint) the PBS would become unsustainable.

By mid 2004, the Labor party was faced with an impending Federal election and had serious trouble costing its tax and spending promises. As a consequence, Labor abandoned their previous principled stand in the Senate of blocking the government's proposed increase in PBS copayments and safety-net thresholds [6] arguing that they needed the additional $1.1 billion to spend on election promises [7]. They also stated that, if returned to government, a substantial proportion of the $1.1 billion might be achieved through administrative reforms to the PBS and savings achieved by the use of cheaper generic drugs as expensive drugs moving off patent. Not surprisingly, consumer and public health groups were appalled with this Labor "back-flip" while the Greens and Democrats said the decision was a disgrace [8]. Labor said the decision was difficult but necessary.

Several other measures were introduced by the government in order to improve the community's understanding of PBS processes and costs. From June 2003, all recommendations of the Pharmaceutical Benefits Advisory Committee (PBAC) to list, not list or defer a decision to list a medicine on the PBS were made publicly available on the PBS website [9]. Unfortunately, only summary information was provided; commercial-in-confidence concerns of pharmaceutical manufacturers precluded making more detailed information available, such as cost-effectiveness data, on which PBAC based its decision.

From 1 August 2003 the full cost of PBS medicines appeared on medicine labels if the price was greater than the co-payment. The full cost included what the consumer has paid and the amount that is paid through the PBS. The aim was to help people understand what medicines really cost and how the PBS helps make medicines affordable for all. In addition, the government commissioned a $24 million advertising campaign that emphasised that patient responsibility was, "the prescription for a healthy PBS". Critics noted that by neglecting to inform the public that pharmaceutical marketing and inappropriate prescribing habits of doctors also produced pressures on the PBS, the campaign missed an opportunity to initiate a more balanced and constructive debate about the viability of the PBS [10].

During the year under review, pharmaceutical reforms designed to stop PBS cost-shifting in Victorian public hospitals were evaluated [11]. The reforms were a joint initiative of Victorian Department of Human Services (DHS) and the Australian Government Department of Health and Ageing (DoHA). Since the early 1990s there had been increasing cost pressures on State and Territory funded public hospitals. Their response included restricting drug supplies to discharged patients, often to only two or three days of treatment. Patients then needed to see their GP to obtain a PBS prescription to cover their needs. The effect was to "cost shift" pharmaceutical supplies from the State and Territories to the Australian Government. The reforms trialed in Victoria allowed public hospital doctors to write PBS prescriptions for both outpatients and discharged inpatients. They also allowed PBS access to a group of cancer chemotherapy drugs for use by day-admitted patients and outpatients. The qualitative evaluation undertaken was generally positive although it noted the reforms had increased the administrative work of both doctors and pharmacists. There was also concern that PBS rules (designed for general practice) were not always appropriate for specialised public hospitals. The Society of Hospital Pharmacists of Australia supported the need to modify PBS procedures to take into account public hospital expertise but also noted the need for more integration of medicines funding [12,13]. Subsequently, the Victorian reforms are being implemented in other States and Territories.

Programs promoting the quality use of medicines (QUM) were further developed throughout 2002–03. Specific programs were coordinated by the National Prescribing Service (NPS), Australian Divisions of General Practice and the Pharmacy Guild of Australia.

The NPS was set up in 1999 with government funding but with an independent Board of Directors in order to provide unbiased educative activities to assist health practitioners (and more recently consumers) to use medicines wisely. Evaluation of NPS activities has consistently shown that spending money on targeted QUM interventions can save considerably more money on the PBS by reducing inappropriate prescribing. It was estimated that NPS activities during the period 1 July 2000 to 30 June 2002 generated PBS savings in the range of $55.6 million to $83.9 million through the following prescribing intervention programs: antibiotics in primary care; peptic ulcer management; management of dyspepsia; COX-2 selective NSAIDs; managing hypertension; and managing dyslipidaemia [14].

In 2003, the NPS received an additional allocation of government money to provide educational material about drugs newly listed on the PBS (the RADAR project). The latter was in response to considerable evidence that intensive pharmaceutical promotion at the time of PBS listing was associated with drugs being prescribed for broader indications than those indicated in the PBS listing (causing so-called PBS "leakage" or "blow-outs") [15]. However, the 2003 NPS educational budget of $12.5 million needs to be compared with the estimated $1.0 billion promotional budget of the Australian pharmaceutical industry [16].

The Enhanced Divisional Quality Use of Medicines (EDQUM) program was a 1999–2000 Federal Budget initiative, originally announced as the Incentives for Quality Prescribing (IQP) program. The program offered Divisions of General Practice (Divisions) a proportion of monies saved if Divisional QUM activities improved prescribing and lowered PBS costs. The Divisions were allowed to use any savings made for a range of primary health care activities. The program evolved significantly due to feedback from the medical profession. It was implemented on a pilot basis in thirteen Divisions on 1 July 2002. Under the program, Divisions were encouraged to invest their own resources in a range of drug utilisation data collection and/or education related activities. Activities were implemented in close consultation with the NPS and focused on one or more of the following target drug groups: antibiotics, peptic ulcer drugs and cardiovascular drugs.

An evaluation of the pilot EDQUM project was undertaken in early 2004 [17]. Barriers to implementation included perceptions that the program was primarily focused on reducing pharmaceutical costs to government; limited capacity of existing prescribing software systems to extract drug utilisation data; and the need for Divisions to take a commercial risk in developing their EDQUM initiatives (due to the absence of up-front funding) which limited their capacity to systematically implement a comprehensive range of strategies. Program achievements included the development of a wide range of shared resource material [18] and the creation (by some Divisions in association with software vendors) of data extraction tools. The latter have allowed a small number of practices to gain access to comprehensive information from which further initiatives to improve quality or change practices can evolve. The evaluation report recommended that standards should be established for prescribing software so that comparable data could be extracted from different systems to facilitate comparison of individual prescribing practice with evidence based guidelines. It also noted the difficulties of attributing any cost-savings in the PBS to Divisional activities. Following this report, the Government supported a four year extension to the EDQUM program in the 2003–2004 Budget.

The Third Community Pharmacy Agreement between the Government and the Pharmacy Guild of Australia (1 July 2000 to 30 June 2005) also provided a range of QUM activities over the year in question including medication reviews of problem patients (conducted at the request of GPs), quality care pharmacy programs and the provision of consumer medicine information [19].

### A potential new threat that emerged

In 2003–2004 the PBS became caught up in negotiations concerning the Australia-United States Free Trade Agreement (AUSFTA). This saga has been extensively reported elsewhere [20,21]. The government remained adamant that the AUSFTA provisions concerning the PBS were benign and would also increase the transparency of PBAC decision-making. Others were concerned that the AUSFTA contained major concessions to the US pharmaceutical industry that undermined the egalitarian principles and operation of the PBS and had the potential to increase the costs of medicinal drugs to Australian consumers. Time will tell who is right.

### Issues still to be tackled

The EDQUM project highlighted the needs for software standards in order to extract comparable drug utilisation data from different prescribing systems. The need for prescribing software standards has also been raised in connection with three other issues of relevance to the PBS: pharmaceutical promotion, independent therapeutic information and drug-drug interaction checking.

The uptake of computers by Australian general practitioners (GPs) was stimulated by the Australian government in 1999. A one-off grant of around $10,000 was offered to those practices that purchased a computer, acquired internet connectivity (an E-mail address) and promised to use computer prescribing software to write the majority of their prescriptions. This increased the numbers of GPs writing prescriptions with the aid of a computer from around 50% in 1999 to more than 90% in 2004 [22]. Legible, printed prescriptions have been one of a number of positive outcomes of this initiative. However, new problems emerged.

One software vendor (Health Communication Network Ltd.) became the dominant market leader because its business model relied on pharmaceutical promotion to heavily subsidise the cost of GPs purchasing and updating its prescribing software (Medical Director™). This business model facilitated software uptake but also resulted in advertisements for the latest and most expensive drugs appearing on the computer screen at the time of prescribing (and elsewhere). GPs using this software package were shown to prescribe more antibiotics per patient than those who wrote 'scripts manually. It was suggested that this may have been due to default settings in the software automatically writing in the maximum number of repeat prescriptions allowed.[23] Another default option in this software was the automatic production of a, "Do not substitute generic drugs" message on the prescription. The latter was eventually changed by the government amending regulation 19(5) of the National Health (Pharmaceutical Benefits) Regulations 1960 [24].

However, the issue of pharmaceutical promotion in prescribing software has yet to be tackled. Pharmaceutical promotion has never been allowed on government supplied 'script pads. It is hard to understand why it was allowed on the computerised equivalent. Pharmaceutical promotion distorts the information flow to physicians by selectively promoting the benefits of the latest and most expensive drugs. It provides minimal information about drug side-effects, contra-indications and opportunity costs. Cost-effective generic drugs are rarely promoted and non-drug solutions usually not at all. Pharmaceutical promotion has clearly been shown to influence physician's prescribing [25] and has resulted in cost-blow outs on the PBS due to "leakage" of prescribing away from cost-effective indications approved by PBAC [26]. In addition, pharmaceutical promotion in prescribing software, occurring at the time of physician decision making, is likely to be much more influential than promotion in medical journals, gimmicks and give-ways. As a consequence, several medical and consumer organisations have advocated further amendment of the National Health (Pharmaceutical Benefits) Regulations 1960, Part V, Regulation 19, to prohibit prescribing software from displaying pharmaceutical advertisements.

Government intervention is also required to ensure that key national resources of objective therapeutic information, such as the Australian Medicines Handbook and Therapeutic Guidelines, are incorporated in prescribing software. The provision of objective therapeutic information is an important strategy of the QUM component of Australian National Medicinal Drug Policy [27]. Ironically, while both the Australian Medicines Handbook and Therapeutic Guidelines have been converted into electronic formats they are not yet included in computerised prescribing software. The problems have included arguments between software vendors and guideline producers over who should pay for the integration and a lack of defined standards for electronic information representation and interfacing.

More recently, the NPS RADAR project has shown the way forward. RADAR provides independent information to health professionals about medicines that have a new or a changed listing on the PBS. RADAR drug monographs have recently been incorporated in four leading GP prescribing packages using an open standard interface. This project has moved ahead because the Australian government provided financial support to both the NPS and software vendors to enable the RADAR integration to take place.

Following a workshop on electronic decision support, HL7 Australia has presented a work plan to the Australian Health Information Council Electronic Decision Support Steering Committee that would build on the RADAR project by incorporating the Australian Medicines Handbook and Therapeutic Guidelines into clinical software in a standard manner [28]. However, this plan has yet to proceed because of a current review of E-Health policy and reorganisation of its governance [29].

The third area of prescribing software requiring government intervention is standards for drug-drug interaction checking. The NPS tested four popular GP software packages by entering a common set of elderly patients on multiple medications [30]. This revealed very different behaviour by different software packages; some missed serious drug-drug interactions, others produced numerous trivial and clinical unimportant alerts. GPs noted that the latter behaviour caused them to turn off all alerts [31]. There is an urgent need for standards concerning acceptable drug-drug interaction detection &/or external assessment of prescribing software, another item on the HL7 Australia work plan.

## Conclusions

The PBS remained in the media and policy spotlight during 2003–04. While the growth rate of the PBS has slowed during the year under review the sustainability of the Scheme remains an ongoing concern. One strategy adopted by the government was to transfer more of the cost of medicines to consumers through higher PBS co-payments and increased safety-net thresholds. However, such measures can result in higher costs elsewhere if poorer patients forgo necessary medicines and end up being hospitalised with uncontrolled disease.

Cost-shifting (and patient inconvenience) was reduced by allowing State and Territory public hospitals limited access to the PBS but these reforms also showed the need for changes in the PBS to make it more suitable for hospital practice and the desirability of further integrating health funding systems.

Educational strategies focusing on the quality use of PBS medicines were successfully pursued but would benefit from increased funding. In addition, there was an exploratory attempt to focus the attention of Divisions on PBS costs by rewarding them with a moiety of any money saved by their members through more cost-effective prescribing. However, the difficulties experienced by the EDQUM project in extracting useful drug utilisation data from computerised prescribing systems highlighted the need for prescribing software standards as did other problems with such software.

Information communication technology and information management (ICT/IM) has the potential to allow individual health practitioners, Divisions and governments to compare what is being done with what is recommended best-practice, highlight major discrepancies, and provide targeted education and appropriate incentives to reduce the gap. However, as the events of 2003–04 show, this potential is unlikely to be realised if the development of clinical computer systems is left solely to market forces.

## Competing interests

Dr. Harvey is a past Board member of Therapeutic Guidelines Limited, Co-Chair of HL7 Australia's Decision Support Technical Committee, a Councillor of the Australia Consumer's Association and a member of the Australian Labor Party.
